# ClipperQTL: ultrafast and powerful eGene identification method

**DOI:** 10.1186/s13059-025-03662-y

**Published:** 2025-07-16

**Authors:** Heather J. Zhou, Xinzhou Ge, Jingyi Jessica Li

**Affiliations:** 1 Department of Statistics and Data Science, University of California, Los Angeles, Los Angeles, CA 90095 USA; 2 Current address: Department of Diabetes and Cancer Metabolism, Beckman Research Institute, City of Hope National Medical Center, Duarte, CA 91010 USA; 3 Current address: Department of Statistics, Oregon State University, Corvallis, OR 97330 USA; 4 Department of Human Genetics, University of California, Los Angeles, Los Angeles, CA 90095 USA; 5 Department of Computational Medicine, University of California, Los Angeles, Los Angeles, CA 90095 USA; 6 Department of Biostatistics, University of California, Los Angeles, Los Angeles, CA 90095 USA; 7 Current address: Biostatistics Program, Public Health Science Division, Fred Hutchinson Cancer Center, Seattle, WA 98109 USA

## Abstract

A central task in expression quantitative trait locus analysis is to identify cis-eGenes, i.e., genes whose expression levels are regulated by at least one local genetic variant. Existing cis-eGene identification methods are either computationally expensive, requiring thousands of permutations per gene (FastQTL), or statistically underpowered (eigenMT and TreeQTL). We propose ClipperQTL, which requires only one permutation for data sets with large sample sizes (>450; ClipperQTL works on smaller data sets too). We show that ClipperQTL performs as well as FastQTL and runs up to 500 times faster. The R package ClipperQTL is available at https://github.com/heatherjzhou/ClipperQTL.

**Supplementary information:**

The online version contains supplementary material available at 10.1186/s13059-025-03662-y.

## Background

Molecular quantitative trait locus (molecular QTL, henceforth “QTL”) analysis investigates the relationship between genetic variants and molecular traits, helping explain the molecular functions of non-coding genetic variants found in genome-wide association studies [1, 2]. Based on the type of molecular phenotype studied, QTL analyses can be categorized into gene expression QTL (eQTL) analyses [3–5], alternative splicing QTL (sQTL) analyses [4], three prime untranslated region alternative polyadenylation QTL (3$'$aQTL) analyses [6], and so on [1, 2]. Among these categories, eQTL analyses, which investigate the association between genetic variants and gene expression levels, are the most common. Therefore, in this work, we focus on eQTL analyses as an example, although everything discussed in this work should in principle apply to other types of QTL analyses as well.

A central task in eQTL analysis is to identify cis-eGenes (henceforth “eGenes”), i.e., genes whose expression levels are regulated by at least one local genetic variant (Problem section). Typically, the genetic variants considered are single nucleotide polymorphisms (SNPs), and “local” means within one megabase of the transcription start site of a gene. This task presents a unique multiple-testing challenge because not only are there many candidate genes, each gene can have up to tens of thousands of local SNPs, and the local SNPs are often in linkage disequilibrium (i.e., associated) with one another.

The eGene identification task is related to but distinct from two other kinds of commonly performed eQTL analysis: a generic analysis that treats every gene-SNP pair as an equal testing unit (e.g., Matrix eQTL [7]), and a fine-mapping analysis that assumes a gene is an eGene and seeks to find out which of its local SNPs are causal (e.g., SuSiE [8]). In practice, a reasonable pipeline would be to first run an eGene identification method and then perform a fine-mapping analysis on each identified eGene [4]. While Matrix eQTL can be naively used to call eGenes (one may simply call as eGenes all genes that appear at least once in the significant gene-SNP pairs; Additional file 1: Section S1.1), it is not designed for this purpose, and both our simulation study (Simulation results section) and Huang et al. [9] show that this naive approach cannot control the false discovery rate (FDR) in the eGene identification problem.

Existing methods that are specifically designed for eGene identification include FastQTL [10], eigenMT [11], and TreeQTL [12]. All three methods share the same two-step approach: first, obtain a gene-level *P* value for each gene; second, apply an FDR control method on the gene-level *P* values to call eGenes. The key difference between the three methods lies in how the gene-level *P* values are obtained.

Among these methods, FastQTL [10] is the most popular. It uses permutations to obtain gene-level *P* values. There are four main ways to use FastQTL, depending on (1) whether the direct or the adaptive permutation scheme is used and (2) whether proportions or beta approximation is used (Table 1). The default way of using FastQTL is to use the adaptive permutation scheme with beta approximation [4, 10]. The adaptive permutation scheme means that the number of permutations is chosen adaptively for each gene (between 1000 and 10,000 by default [4, 10]); beta approximation helps produce higher-resolution gene-level *P* values given the number of permutations (Additional file 1: Section S1.2). The main drawback of FastQTL is the lack of computational efficiency, since it requires thousands of permutations for each gene. A faster implementation of FastQTL named tensorQTL has been developed [13], but it relies on graphics processing units (GPUs), which are more expensive than central processing units (CPUs) and not universally available.

**Table 1:** Summary of the eGene identification methods we compare

	Method category	Method	Note	Method name for speed comparison
	(A)	(B)	(C)	(D)
1	Matrix eQTL	Matrix eQTL		
2	FastQTL	FastQTL_1K-10K_prop		FastQTL_1K-10K
3		FastQTL_1K-10K_beta	Default FastQTL method	FastQTL_1K-10K
4		FastQTL_1K_prop		FastQTL_1K
5		FastQTL_1K_beta		FastQTL_1K
6	tensorQTL	tensorQTL_10K_beta	Default tensorQTL method	tensorQTL_10K
7	eigenMT	eigenMT		eigenMT
8	TreeQTL	TreeQTL_BY	Default TreeQTL method	TreeQTL
9		TreeQTL_Storey		TreeQTL
10	ClipperQTL	ClipperQTL_standard_1K		ClipperQTL_standard_1K
11		ClipperQTL_Clipper_1		ClipperQTL_Clipper_1
12		ClipperQTL_Clipper_20		ClipperQTL_Clipper_20

Details of these methods can be found in the ClipperQTL section and Additional file 1: Section S1

**Fig. 1:**
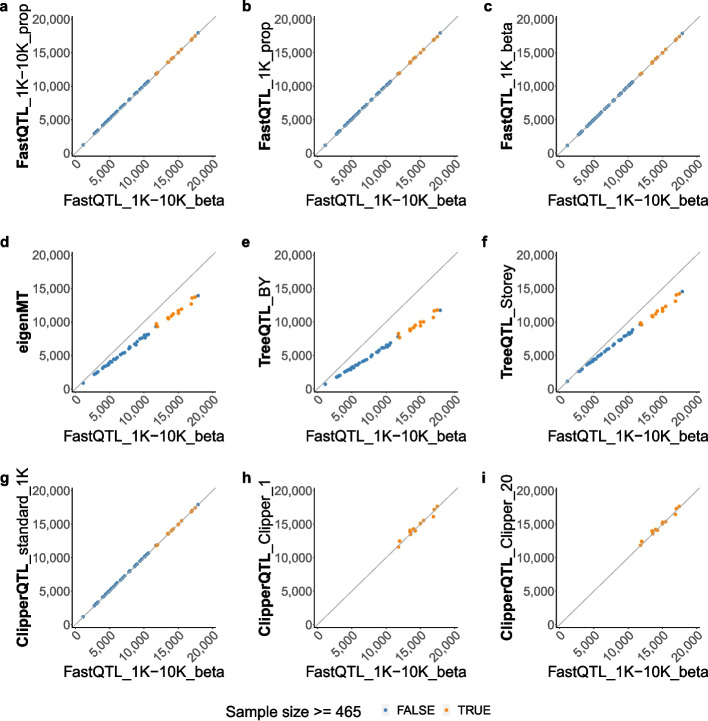
Number of eGenes comparison based on GTEx bulk data [4] (Table 1; see Data preparation and analysis section for the analysis details). Each dot corresponds to a tissue. The *x*-axis and *y*-axis both represent numbers of eGenes identified by different methods. Diagonal lines through the origin are shown to help with visualization. **a–c** The four variants of FastQTL identify almost the same numbers of eGenes as one another. **d–f** eigenMT and TreeQTL methods identify fewer eGenes than FastQTL. **g–i** ClipperQTL methods identify almost the same numbers of eGenes as FastQTL in tissues with the appropriate sample sizes (ClipperQTL section). We use 465 as the sample size cutoff because the next largest sample size is 396. See Additional file 1: Fig. S2 for an analysis of the overlap between identified eGenes

eigenMT [11] and TreeQTL [12] have been proposed as faster alternatives to FastQTL. Neither method uses permutations. In a nutshell, eigenMT uses Bonferroni correction to calculate a gene-level *P* value for each gene, but instead of using the *actual* numbers of local SNPs, it estimates the *effective* number of local SNPs for each gene by performing a principal-component-like analysis (Additional file 1: Section S1.3). This is done for better power because the *actual* number of local SNPs for a gene is often substantially greater than the *effective* number of local SNPs due to linkage disequilibrium. TreeQTL, on the other hand, uses Simes’ rule [14] to calculate a gene-level *P* value for each gene (Additional file 1: Section S1.4). Our analysis shows that both eigenMT and TreeQTL have lower power than FastQTL (Figs. 1 and 4).

Clipper [15] is a *P* value-free FDR control method. Given a large number of features (e.g., genes), a number of measurements under the experimental (e.g., treatment) condition, and a number of measurements under the background (e.g., control) condition, Clipper works as the following: first, obtain a contrast score for each feature based on the experimental and background measurements (for example, the contrast score may be the average of the experimental measurements minus the average of the background measurements); second, given a target FDR (e.g., 0.05), obtain a cutoff for the contrast scores; lastly, call the features with contrast scores above the cutoff as discoveries. The idea is that the contrast scores of the uninteresting features (e.g., genes whose expected expression levels are *not* increased by the treatment) will be roughly symmetrically distributed around zero, and the outlying contrast scores in the right tail likely belong to interesting features. Notably, Clipper produces a *q* value for each feature (similar to Storey’s *q* values [16]), so that the features can be ranked from the most significant to the least significant.

In this work, we propose ClipperQTL for eGene identification, which reduces the number of permutations needed from thousands to one for data sets with large sample sizes ($>450$) by using the contrastive strategy developed in Clipper; for data sets with smaller sample sizes, ClipperQTL uses the same permutation-based approach as FastQTL. Using GTEx bulk RNA-seq data [4], OneK1K single-cell RNA-seq data [5], and simulated data, we show that ClipperQTL performs as well as FastQTL and runs up to 500 times faster if the contrastive strategy is used and 50 times faster if the conventional permutation-based approach is used (we refer to the two variants of ClipperQTL as the Clipper variant and the standard variant, respectively; ClipperQTL section). ClipperQTL does not rely on GPUs, but it is still up to 30 times more computationally efficient than tensorQTL, a GPU-based implementation of FastQTL.

## Results

### Real data results

We compare the performance and run time of different variants of FastQTL, eigenMT, TreeQTL, and ClipperQTL (Table 1) on both GTEx bulk [4] and OneK1K single-cell [5] expression data. The data preparation and analysis details are described in the Data preparation and analysis section. Following standard practice [5, 17], we analyze the single-cell data in a pseudo-bulk manner. The GTEx data contains 49 individual-by-gene expression matrices, one for each tissue; the sample sizes (numbers of individuals) range from 73 to 706, and the numbers of genes range from 20,315 to 26,854 (except that testis has 35,007 genes). The OneK1K data contains 12 individual-by-gene expression matrices, one for each cell type; the sample sizes range from 933 to 981, and the numbers of genes range from 477 to 9643. That is, the OneK1K expression matrices have larger sample sizes but smaller numbers of genes than the GTEx expression matrices (due to zeros in the single-cell count data; Data preparation and analysis section). We do not include Matrix eQTL [7] in our real data comparison because both our simulation study (Simulation results section) and Huang et al. [9] show that Matrix eQTL cannot control the FDR in the eGene identification problem.

**Fig. 2:**
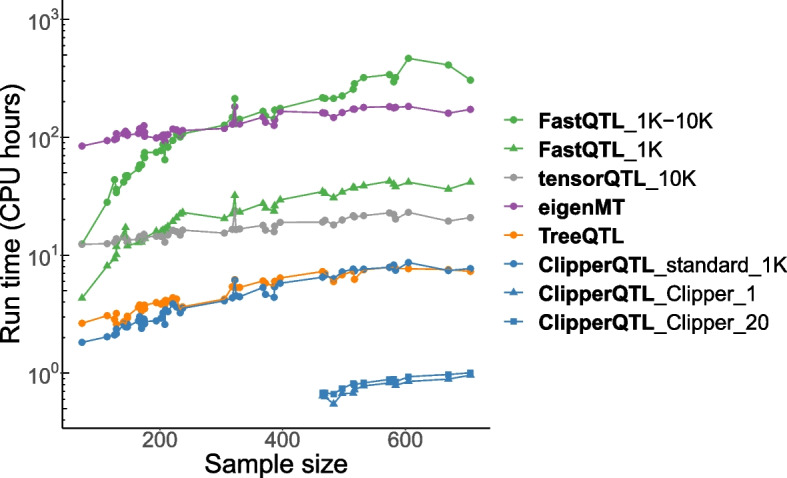
Run time comparison based on GTEx bulk data [4] (Table 1; see Data preparation and analysis section for the analysis details). Each dot corresponds to a tissue. FastQTL_1K-10K takes under 500 CPU hours. ClipperQTL_standard_1K takes under 10 CPU hours. ClipperQTL_Clipper_1 and ClipperQTL_Clipper_20 take under 1 CPU hour. Run times of ClipperQTL_Clipper_1 and ClipperQTL_Clipper_20 are only shown for tissues with sample sizes $\ge 465$ (Fig. 1). The GPU run time of tensorQTL is converted to CPU run time by a factor of 20 based on the current relative costs of GPUs vs. CPUs on Amazon Web Services (AWS)

The results from the GTEx data are summarized in Figs. 1 and 2 and Additional file 1: Fig. S2. We find that the four variants of FastQTL produce almost identical results as one another. Specifically, the numbers of eGenes identified by the four methods are almost identical (Fig. 1), and the identified eGenes highly overlap (Additional file 1: Fig. S2). This means the adaptive permutation scheme and beta approximation of FastQTL (Additional file 1: Section S1.2) are not critical to the performance of FastQTL; the simplest variant, FastQTL_1K_prop, is sufficient. To the best of our knowledge, this is the first time that this has been discovered in the literature. Further, we find that eigenMT and TreeQTL methods identify fewer eGenes than FastQTL (Fig. 1). In contrast, ClipperQTL methods produce almost identical results as FastQTL in tissues with the appropriate sample sizes (ClipperQTL section; Fig. 1 and Additional file 1: Fig. S2). In terms of run time comparison (Fig. 2), we find that eigenMT has little computational advantage over FastQTL, and TreeQTL has no computational advantage over the standard variant of ClipperQTL (which is slower than the Clipper variant of ClipperQTL). Both the standard variant and the Clipper variant of ClipperQTL are orders of magnitude faster than FastQTL. In particular, the standard variant of ClipperQTL is about five times faster than FastQTL_1K_prop—the simplest FastQTL method—even though the algorithms are equivalent (ClipperQTL section); we attribute this to differences in software implementation (for example, we aggregate correlations of vectors into correlations of matrices). Compared to the default FastQTL method, the standard variant and the Clipper variant of ClipperQTL are about 50 times and 500 times faster, respectively. In addition, ClipperQTL is up to 30 times more computationally efficient than tensorQTL, a GPU-based implementation of FastQTL (Fig. 2).

**Fig. 3:**
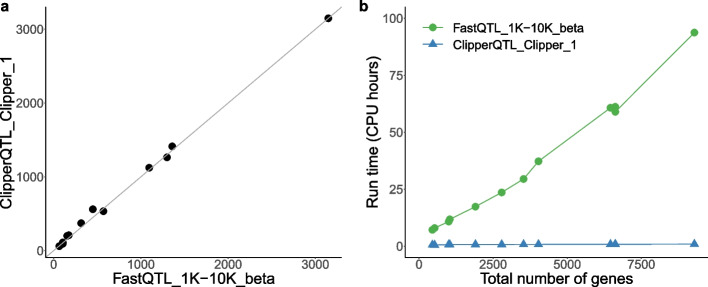
Number of eGenes and run time comparison based on OneK1K single-cell data [5] (Table 1; see Data preparation and analysis section for the analysis details). Each dot corresponds to a cell type. **a** The *x*-axis and *y*-axis both represent numbers of eGenes identified. A diagonal line through the origin is shown to help with visualization. ClipperQTL_Clipper_1 identifies almost the same numbers of eGenes as FastQTL_1K-10K_beta. The overlap between the identified eGenes averages at about 98% (see Additional file 1: Fig. S2 for our definition of overlap). Replacing ClipperQTL_Clipper_1 with ClipperQTL_Clipper_20 or ClipperQTL_Clipper_100 would yield very similar plots (not shown). **b** FastQTL_1K-10K_beta takes under 100 CPU hours. ClipperQTL_Clipper_1 takes under 1 CPU hour

The results from the OneK1K data (Fig. 3) confirm our findings from the GTEx data. When the sample size is large enough (which is the case in the OneK1K data), the Clipper variant of ClipperQTL with only one permutation produces nearly identical results as FastQTL and takes less than one-hundredth of the time to run. Since the run time of FastQTL grows at least linearly with the total number of genes and the run time of ClipperQTL is nearly constant (Fig. 3b), we believe that the computational advantage of ClipperQTL would have been even more substantial if more genes had remained after filtering in the OneK1K data (Data preparation and analysis section).

### Simulation results

**Fig. 4:**
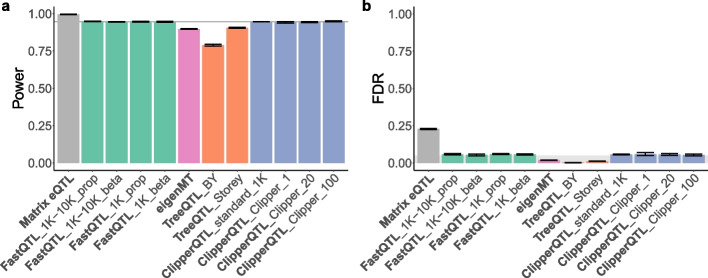
Power and FDR comparison based on simulation study (Table 1; Simulation results section). The target FDR is set at 0.05 (gray shaded area in **b**). The height of each bar represents the average across all simulated data sets. Error bars indicate standard errors. In **a**, a horizontal line at the height of the bar for FastQTL_1K-10K_beta is shown to help with visualization. All methods except Matrix eQTL can approximately control the FDR. FastQTL and ClipperQTL methods have higher power than eigenMT and TreeQTL methods

In our simulation study, we approximately follow the data simulation in the second, more realistic simulation design of Zhou et al. [18], which approximately follows the data simulation in Wang et al. [8]. We simulate three data sets in total. Each data set is simulated according to Additional file 1: Algorithm S5 with sample size $n=838$, number of genes $p=1000$, number of covariates $\widetilde{K}=20$, proportion of variance explained by genotype in eGenes $\texttt {PVEGenotype}=0.02$ (cis effect only), and proportion of variance explained by covariates $\texttt {PVECovariates}=0.5$. $\texttt {PVEGenotype}=0.02$ is in line with the settings in Zhou et al. [18] and Wang et al. [8]; a low PVEGenotype helps differentiate the different methods in terms of power (Fig. 4a). All covariates are assumed to be known covariates.

The results from our simulation study are summarized in Fig. 4. We confirm the finding in Huang et al. [9] that Matrix eQTL cannot control the FDR in the eGene identification problem. All other methods can approximately control the FDR. Further, FastQTL and ClipperQTL methods have higher power than eigenMT and TreeQTL methods, consistent with our real data results (Real data results section).

## Discussion

We have shown that ClipperQTL performs as well as FastQTL and runs up to 500 times faster. Without relying on GPUs, it is up to 30 times more computationally efficient than tensorQTL, a GPU-based implementation of FastQTL.

We propose two main variants of ClipperQTL: the standard variant and the Clipper variant. The standard variant is equivalent to FastQTL with the direct permutation scheme and proportions (Additional file 1: Algorithm S1) and is suitable for a wide range of sample sizes. The Clipper variant uses the contrastive strategy developed in Clipper [15] (Algorithm 1) and is only recommended for data sets with large sample sizes ($>450$).

Regarding which variant of ClipperQTL should be used when the sample size is large enough ($>450$), we believe that if computational efficiency is a priority, then the Clipper variant should be used. However, if the majority of data sets in the study have smaller sample sizes, then the researcher may choose to use the standard variant on all data sets for consistency.

A possible extension of ClipperQTL lies in trans-eGene identification. Compared to cis-eGenes, trans-eGenes are currently identified in very small numbers [4], possibly due to the lack of power of existing approaches. FastQTL currently cannot be used to identify trans-eGenes, likely because of the computational burden. Since the Clipper variant of ClipperQTL needs very few permutations, it is much more suitable for trans-eGene identification than FastQTL. We believe that the current framework of ClipperQTL could be directly applicable to trans-eGene identification.

The computational efficiency of ClipperQTL comes from three levels. First, due to the overlap of local common SNPs across genes, both FastQTL and ClipperQTL make use of the one-to-one correspondence between the absolute value of partial correlation and the *P* value of the variable of interest in linear models (Additional file 1: Section S1.1) to substantially reduce the number of linear models that need to be fitted. Second, ClipperQTL has software implementation advantages over FastQTL; for example, it aggregates correlations of vectors into correlations of matrices, which is significantly more computationally efficient (Real data results section). Third, the Clipper variant of ClipperQTL requires orders of magnitude fewer permutations than FastQTL (ClipperQTL section).

Although existing eGene identification methods use linear models and do not account for related individuals with linear mixed models (LMMs; as is done in genome-wide association studies [19]), in principle, both variants of ClipperQTL can be extended to LMMs (instead of taking *P* values from linear models, one may simply take *P* values from LMMs). However, since the computational efficiency of both variants of ClipperQTL relies heavily on the one-to-one correspondence between the absolute value of partial correlation and the *P* value of the variable of interest in linear models (as discussed in the previous paragraph) and there may not be an equivalent one-to-one correspondence in LMMs, the computational burden that comes with LMMs may be a challenge. Emerging single-cell-specific eGene identification methods such as SAIGE-QTL [20] use generalized linear mixed models (GLMMs; for example, Poisson GLMMs) to account for related cells (i.e., cells that come from the same individuals). In principle, the standard variant of ClipperQTL can be extended to GLMMs, similar to how it can be extended to LMMs. However, the computational burden may be a challenge. On the other hand, the Clipper variant of ClipperQTL constructs null data sets using permutation after residualization, which may not be applicable in GLMMs because the residuals would not be counts and thus may not be suitable response variables in GLMMs. An alternative model-based approach for generating the null data may be appropriate [21]. We leave these questions for future research.

## Conclusions

Our work demonstrates the potential of the contrastive strategy developed in Clipper [15] and provides a simpler and more efficient way of identifying cis-eGenes. The R package ClipperQTL is available at https://github.com/heatherjzhou/ClipperQTL.

## Methods

### Problem

Here we describe the eGene identification problem and introduce the notations for this work.

The input data are as follows. Let *Y* denote the $n\times p$ fully processed gene expression matrix with *n* individuals and *p* genes. For gene $j\,$, $j=1,\cdots ,p\,$, the relevant genotype data is stored in $S_j\,$, the $n\times q_j$ genotype matrix, where each column of $S_j$ corresponds to a local common SNP for gene $j\,$ (conceptually speaking; in reality, all genotype data may be stored in one file). Let *X* denote the $n\times K$ covariate matrix with *K* covariates. Using our analysis of GTEx’s Colon - Transverse data [4] (Data preparation and analysis section) as an example, we have $n=368$, $p=25{,}379$, $q_j$ typically under 15,000, and $K=37$, including eight known covariates and 29 inferred covariates (Data preparation and analysis section).

The assumption is that for $j=1,\cdots ,p\,$, $Y[\,,\,j]\,$, the *j*th column of *Y*, is a realization of the following random vector:1where $\mathbbm{1}$ denotes the $n\times 1$ matrix of ones, $S_j$ is defined as above, $\widetilde{X}$ is the true covariate matrix (which *X* tries to capture), all entries of $\beta _{0j}\,$, $\beta _{1j}\,$, and $\beta _{2j}$ are fixed but unknown parameters, and $\epsilon _j$ is the random noise. In particular, it is assumed that at most a small number of entries of $\beta _{1j}$ are nonzero [8]. If all entries of $\beta _{1j}$ are zero, then gene *j* *is not* an eGene. On the other hand, if at least one entry of $\beta _{1j}$ is nonzero, then gene *j* *is* an eGene. The goal is to identify which of the *p* genes are eGenes given *Y*, $\left\{ S_j\right\} _{j=1}^p\,$, and *X*.

### ClipperQTL

We propose two main variants of ClipperQTL: the standard variant and the Clipper variant. The standard variant is equivalent to FastQTL with the direct permutation scheme and proportions (Additional file 1: Algorithm S1) and is suitable for a wide range of sample sizes. The Clipper variant uses the contrastive strategy developed in Clipper [15] (Algorithm 1) and is only recommended for data sets with large sample sizes ($>450$). The development of ClipperQTL is discussed in Additional file 1: Section S3. A key technical difference between the standard variant and the Clipper variant is that in the standard variant, gene expression is permuted first and then residualized (following FastQTL; Additional file 1: Algorithm S1), whereas in the Clipper variant, gene expression is residualized first and then permuted (based on empirical evidence).

The main input parameter of ClipperQTL under both variants is *B*, the number of permutations. For the standard variant, *B* is set at 1000 by default. For the Clipper variant, we recommend setting $B=1$ or *B* between 20 and 100 (Additional file 1: Section S3). The result of the Clipper variant is robust to the choice of *B* as long as *B* is one of the recommended values (Additional file 1: Figs. S3 and S4). The computational complexity of both variants of ClipperQTL is $O(Bp\bar{q}n)$, where $\bar{q}$ denotes $\frac{1}{p}\sum _{j=1}^p q_j$, the average of $q_j$. This is the same computational complexity as that of FastQTL, but due to implementation advantages (Real data results section) and the fact that *B* can be much smaller when the sample size is large enough ($>450$), ClipperQTL is much faster in practice (Figs. 2 and 3b).

**Figure:**
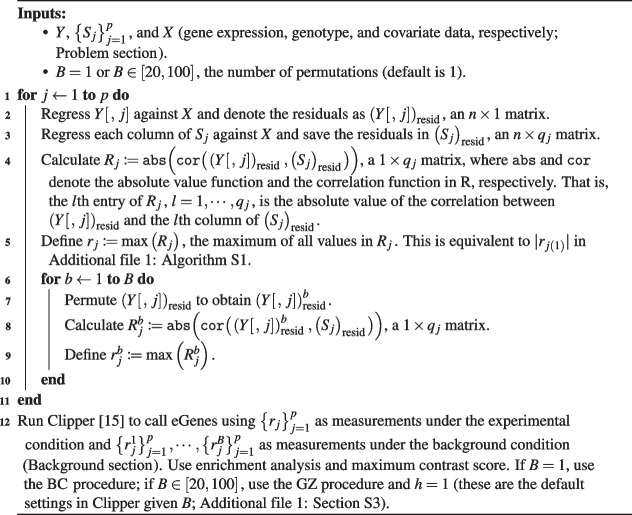
**Algorithm 1** The Clipper variant of ClipperQTL

### Data preparation and analysis

Here we describe how we prepare and analyze the GTEx bulk [4] and OneK1K single-cell [5] expression data.

For the GTEx bulk expression data, we start with the 49 fully processed gene-by-individual expression matrices, one for each tissue. For each gene, we consider SNPs within one megabase (Mb) of the transcription start site (TSS) of the gene [4]; we use 0.01 as the threshold for the minor allele frequency (MAF) of a SNP and 10 as the threshold for the number of individuals with at least one copy of the minor allele (MA samples) [10]. We include eight known covariates and a number of top expression PCs (principal components) as inferred covariates [18]. The eight known covariates are the top five genotype PCs, WGS sequencing platform (HiSeq 2000 or HiSeq X), WGS library construction protocol (PCR-based or PCR-free), and donor sex [4]. The number of expression PCs is chosen via the Buja and Eyuboglu (BE) algorithm [18, 22] for each tissue. The target FDR for eGene identification is set at 0.05.

For the OneK1K single-cell expression data, we start with the gene-by-cell count matrix (32,738 genes by 1,272,489 cells), each cell belonging to one of 981 individuals. We perform normalization and averaging using the NormalizeData and AverageExpression functions in Seurat [23] (the default settings are used). That is, first, we normalize each count as $\texttt {log}(\frac{\text {count}}{\text {per-cell total}}\times 10{,}000+1)$, where log represents the natural logarithm function. This step does not change the dimensions of the count matrix. Then, for each of the 16 cell types [5], we take the average per gene-individual combination in the non-log space (i.e., after exponentiating and subtracting one), obtaining a gene-by-individual matrix. Finally, for each cell type, we only keep genes with nonzero expression in at least 90% of individuals [5]. This leaves us with 12 cell types with at least one gene (that is, four cell types have zero genes remaining after filtering). Given the 12 gene-by-individual expression matrices, our data analysis protocol (including genotype QC) is identical to that described in the previous paragraph, except that we use two known covariates (sex and age [5]), and the number of expression PCs is chosen via the elbow method [18]. The reason why we use the BE algorithm for the GTEx data but the elbow method for the OneK1K data is because we find that in our simulated data (Additional file 1: Section S2), the BE algorithm can recover the true number of covariates well. However, while the numbers of PCs chosen by the BE algorithm seem reasonable in the GTEx data (between 12 and 56), the numbers of PCs chosen by the BE algorithm in the OneK1K data are too high (between 58 and 159; in contrast, the elbow method chooses between 19 and 68 PCs).

## Supplementary information

Additional file 1: Supplementary materials. Includes all supplementary text, figures, tables, and algorithms.

## Data Availability

The R package ClipperQTL is available at https://github.com/heatherjzhou/ClipperQTL [24] (GPL-3.0 license). The code used to generate the results in this work is available at 10.5281/zenodo.8259928 [25] (MIT license). In addition, this work makes use of the following data and software: • GTEx V8 public data, including fully processed gene expression matrices and known covariates, are downloaded from https://gtexportal.org/home/datasets. • GTEx V8 protected data, specifically, the whole genome sequencing (WGS) phased genotype data, are downloaded from the AnVIL repository with an approved dbGaP application (see https://gtexportal.org/home/protectedDataAccess). • OneK1K data, including single-cell count data, cell type annotation, genotype data, and known covariate data, are generously provided by the authors. • FastQTL (https://github.com/francois-a/fastqtl, accessed October 29, 2020). • tensorQTL (https://github.com/broadinstitute/tensorqtl, accessed April 16, 2024). • Matrix eQTL R package Version 2.3 (https://cran.r-project.org/web/packages/MatrixEQTL, accessed March 6, 2023). • eigenMT (https://github.com/joed3/eigenMT, accessed March 6, 2023). • TreeQTL R package Version 2.0 (https://bioinformatics.org/treeqtl, accessed March 6, 2023).
