# Supplementary material for: ClipperQTL: ultrafast and powerful eGene identification method
Source: Genome Biol. 2025 Jul 16;26:207. doi: 10.1186/s13059-025-03662-y (PMC12265108; doi:10.1186/s13059-025-03662-y)
Supplement: Supplementary file 1 — Additional file 1: Supplementary materials. Includes all supplementary text, figures, tables, and algorithms. [file 13059_2025_3662_MOESM1_ESM.pdf]

Supplementary materials for

ClipperQTL: ultrafast and powerful eGene identification method

Heather J. Zhou, Xinzhou Ge, Jingyi Jessica Li

S1 Existing eGene identification methods

In this section, we review the existing eGene identification methods and describe the variants that we compare in this work (Table 1).

Recall the notations from Section 5.1. Let Y denote the $n \times p$ fully processed gene expression matrix with n individuals and p genes. For gene j, $j = 1, \cdots, p$, the relevant genotype data is stored in S_j, the $n \times q_j$ genotype matrix, where each column of S_j corresponds to a local common SNP for gene j. Let X denote the $n \times K$ covariate matrix with K covariates.

S1.1 Matrix eQTL

Conceptually speaking, Matrix eQTL [1] works as follows: for $j = 1, \cdots, p$, $l = 1, \cdots, q_j$, run the linear regression represented by the following R `lm()` formula:

$$Y[\,,j] \sim S_j[\,,l] + X \tag{S1}$$

and obtain the P-value for the null hypothesis that the coefficient corresponding to $S_j[\,,l]$ is zero given the covariates; denote this P-value as p_{jl}. Therefore, a total of $\sum_{j=1}^{p} q_j$ P-values are obtained, one for each gene-SNP pair. Matrix eQTL then uses the Benjamini-Hochberg (BH) procedure [2] on these P-values to call significant gene-SNP pairs [1]. To call eGenes using Matrix eQTL in this work, we call as eGenes all genes that appear at least once in the significant gene-SNP pairs.

In reality, Matrix eQTL uses the following equivalent approach to obtain the P-values, which is more computationally efficient due to the overlap of local common SNPs across genes. For $j = 1, \cdots, p$, $l = 1, \cdots, q_j$, first, regress the gene expression against the covariates:

$$Y[\,,j] \sim X. \tag{S2}$$

Second, regress the genotype against the covariates:

$$S_j[\,,l] \sim X. \tag{S3}$$

Then, calculate the Pearson correlation between the expression residuals from (S2) and the genotype residuals from (S3) and denote it as r_{jl}. This is the partial correlation between $Y[\,,j]$ and $S_j[\,,l]$ conditional on X.

Lastly, convert the partial correlation to a test statistic using

$$t_{jl} = r_{jl}\sqrt{\frac{n-2-K}{1-r_{jl}^2}} \tag{S4}$$

and convert the test statistic to a P-value using

$$p_{jl} = 2 \times \mathbb{P}\left(T \geq |t_{jl}|\right),\ T \sim t_{n-2-K}, \tag{S5}$$

where $\mathbb{P}$ denotes probability, $|t_{jl}|$ denotes the absolute value of t_{jl}, and T is a random variable following the t-distribution with $n-2-K$ degrees of freedom.

Notably, the larger $|r_{jl}|$ (the absolute value of r_{jl}), the smaller p_{jl}, and vice versa. Both FastQTL (the variants using proportions; Section S1.2) and ClipperQTL (the standard variant; Section 5.2) make use of this fact.

S1.2 FastQTL

There are four main ways to use FastQTL [3], depending on (1) whether the direct or the adaptive permutation scheme is used and (2) whether proportions or beta approximation is used. The direct permutation scheme with either proportions or beta approximation is summarized in Algorithm S1. The adaptive permutation scheme is identical except the number of permutations is chosen adaptively between B_{min} and B_{max} (two input parameters) for each gene rather than directly inputted (see Ongen et al. [3] for details).

The default way of using FastQTL is to use the adaptive permutation scheme ($B_{\text{min}} = 1000$ and $B_{\text{max}} = 10{,}000$) with beta approximation [3, 4]. In total, we compare four ways of using FastQTL in this work including the default approach: FastQTL_1K-10K_prop, FastQTL_1K-10K_beta (the default), FastQTL_1K_prop, and FastQTL_1K_beta (see Table 1). That is, the number of permutations is either fixed at 1000 or chosen adaptively between 1000 and 10,000 for each gene, and either proportions or beta approximation is used.

In addition to identifying eGenes, FastQTL can also output significant gene-SNP pairs. We summarize the algorithm for this in Algorithm S2.

Algorithm S1: The direct permutation scheme of FastQTL

Inputs:

- Y, $\{S_j\}_{j=1}^{p}$, and X (gene expression, genotype, and covariate data, respectively; Section 5.1).
- B, the number of permutations.

1 **for** $j \leftarrow 1$ **to** p **do**
2   Obtain $r_{j1}, \cdots, r_{jq_j}$, the partial correlation between $Y[\,,j]$ and $S_j[\,,1], \cdots, S_j[\,,q_j]$ (respectively) conditional on X (Section S1.1).
3   Denote the one with the largest absolute value as $r_{j(1)}$.
4   **for** $b \leftarrow 1$ **to** B **do**
5   Permute $Y[\,,j]$ (leave S_j and X unchanged).
6   Obtain $r^b_{j1}, \cdots, r^b_{jq_j}$, the partial correlation between $Y[\,,j]$ after permutation and $S_j[\,,1], \cdots, S_j[\,,q_j]$ (respectively) conditional on X.   // Analogous to Line 2.
7   Denote the one with the largest absolute value as $r^b_{j(1)}$.   // Analogous to Line 3.
8   **end**
9   **if** *using proportions* **then**
10         $\widetilde{p}_j$, the gene-level P-value for gene j, is defined as $\widetilde{p}_j := \left(\sum_{b=1}^{B} \mathbb{1}\{|r^b_{j(1)}| \geq |r_{j(1)}|\} + 1\right) / (B+1)$.   // Roughly the proportion of permutations with more extreme outcomes. The addition of one in the numerator and the denominator helps avoid P-values that are exactly zero.
11   **else if** *using beta approximation* **then**
12   Find $true\_df \in (0, \infty)$, which is to replace $n-2-K$ when converting $r_{j(1)}$ and $\{r^b_{j(1)}\}_{b=1}^{B}$ to P-values using (S4) and (S5).   // See the source code of FastQTL for how $true\_df$ is defined. In a nutshell, $true\_df$ minimizes the absolute difference between 1 and the method of moments estimate for the first shape parameter of the beta distribution from the P-values.
13   Convert $r_{j(1)}$ and $\{r^b_{j(1)}\}_{b=1}^{B}$ to P-values using (S4) and (S5) with $n-2-K$ replaced by $true\_df$. Denote these P-values as $p_{j(1)}$ and $\{p^b_{j(1)}\}_{b=1}^{B}$.
14   Fit a beta distribution to $\{p^b_{j(1)}\}_{b=1}^{B}$ using maximum likelihood estimation. Denote the cumulative distribution function of the fitted beta distribution as F_j.
15         $\widetilde{p}_j$, the gene-level P-value for gene j, is defined as $\widetilde{p}_j := F_j\left(p_{j(1)}\right)$.
16   **end**
17 **end**
18 Use Storey's q-value [5] on $\{\widetilde{p}_j\}_{j=1}^{p}$ to call eGenes.

Algorithm S2: Identification of significant gene-SNP pairs in FastQTL

Input:

- Intermediate and final results from Algorithm S1 using either the direct or the adaptive permutation scheme *and* beta approximation (rather than proportions).

Define p_t (following the notation of the GTEx Consortium [4]) as the average of the gene-level P-value of the most significant non-eGene and the gene-level P-value of the least significant eGene (see the source code of FastQTL). That is, p_t is defined as the average of two of $\widetilde{p}_1, \cdots, \widetilde{p}_p$.

for $j \leftarrow 1$ **to** p **do**

  if *gene j is identified as an eGene* **then**

    Define $threshold_j := F_j^{-1}(p_t)$, where F_j^{-1} denotes the inverse function of F_j.

    Convert $r_{j1}, \cdots, r_{jq_j}$ (Line 2 of Algorithm S1) to P-values using (S4) and (S5) (without replacing $n-2-K$ with $true\_df$).

    If the P-value corresponding to a SNP is less than or equal to $threshold_j$, then gene j and this SNP are together identified as a significant gene-SNP pair.

  end

end

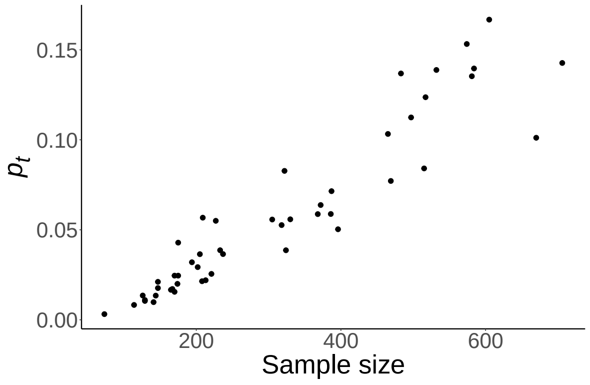

Figure S1: Scatter plot of p_t (Algorithm S2) from FastQTL_1K-10K_beta versus sample size in GTEx bulk data [4] (see Section 5.3 for the analysis details). This scatter plot contains 49 dots, each corresponding to a tissue. We see that p_t increases roughly linearly with sample size.

S1.3 eigenMT

We summarize eigenMT [6] in Algorithm S3. After obtaining the gene-level P-values, eigenMT does not specify what method to use to control the false discovery rate when calling eGenes. Therefore, in this work, we use Storey's q-value [5] following FastQTL (Section S1.2). In addition to producing the gene-level P-values, eigenMT also outputs the most significantly associated SNP for each gene.

Algorithm S3: eigenMT

Inputs:

- Results from Matrix eQTL, i.e., p_{jl}, $j = 1, \cdots, p$, $l = 1, \cdots, q_j$ (Section S1.1).
- $\{S_j\}_{j=1}^{p}$, genotype data (Section 5.1).
- W, window size for partitioning genotype data (default is 200).
- C, threshold for the cumulative proportion of variance explained (default is 0.99).

for $j \leftarrow 1$ **to** p **do**
  Consider $p_{j1}, \cdots, p_{jq_j}$. Denote the smallest one as $p_{j(1)}$.
  Initialize q_j^{eff}, the effective number of local common SNPs for gene j, at 0. The goal is to calculate q_j^{eff} from S_j.
  Break S_j vertically into $\lceil \frac{q_j}{W} \rceil$ chunks, each chunk an $n \times W$ matrix except the last chunk, which may have fewer columns.
  for $i \leftarrow 1$ **to** $\lceil \frac{q_j}{W} \rceil$ **do**
    Denote the number of columns in the ith chunk of S_j as W_{ji} (if $i < \lceil \frac{q_j}{W} \rceil$, then $W_{ji} = W$).
    if $W_{ji} = 1$ **then**
      $q_j^{\text{eff}} \leftarrow q_j^{\text{eff}} + 1$.
    else
      Obtain the Ledoit-Wolf estimate [7] of the covariance matrix of the ith chunk of S_j using `sklearn.covariance.LedoitWolf()` in Python.
      Convert the estimated covariance matrix to a correlation matrix.
      Obtain the eigenvalues of the correlation matrix, $\lambda_1 \geq \cdots \geq \lambda_{W_{ji}}$, using `scipy.linalg.eigvalsh()` in Python.
      Set all negative eigenvalues (if any) to 0. `// See the source code of eigenMT.`
      $q_j^{\text{eff}} \leftarrow q_j^{\text{eff}} + \arg\min_K \left(\frac{\sum_{k=1}^{K} \lambda_k}{W_{ji}} \geq C \right)$. That is, increment q_j^{eff} by the minimum number of top eigenvalues required to pass the threshold for the cumulative proportion of variance explained. `// See the source code of eigenMT.`
    end
  end
  $\widetilde{p}_j$, the gene-level P-value for gene j, is defined as $\widetilde{p}_j := \min\left(p_{j(1)} \times q_j^{\text{eff}}, 1 \right)$.
end

S1.4 TreeQTL

TreeQTL [8] uses Simes' rule [9] to calculate a gene-level P-value for each gene (Algorithm S4). After obtaining the gene-level P-values, TreeQTL allows the user to use Bonferroni correction, BH [2], or Benjamini-Yekutieli (BY) [10] to call eGenes (the default is BY).

We compare two variants of TreeQTL in this work: TreeQTL_BY (the default) and TreeQTL_Storey (see Table 1). In TreeQTL_Storey, we use Storey's q-value [5] on the gene-level P-values to call eGenes, following FastQTL (Section S1.2). We do not include variants of TreeQTL using Bonferroni correction or BH in our comparison because Bonferroni correction

aims to control the family-wise error rate rather than the false discovery rate, and BH is more stringent than Storey's q-value (we show that even TreeQTL_Storey has lower power than FastQTL; Figures 1 and 4).

Algorithm S4: TreeQTL

Input:

- Results from Matrix eQTL, i.e., p_{jl}, $j = 1, \cdots, p$, $l = 1, \cdots, q_j$ (Section S1.1).

for $j \leftarrow 1$ **to** p **do**
  Consider $p_{j1}, \cdots, p_{jq_j}$. Denote the order statistics as $p_{j(1)}, \cdots, p_{j(q_j)}$, with $p_{j(1)}$ being the smallest and $p_{j(q_j)}$ being the largest.
  $\widetilde{p}_j$, the gene-level P-value for gene j, is defined as $\widetilde{p}_j := \min_{l=1,\cdots,q_j} p_{j(l)} \frac{q_j}{l}$, following Simes' rule [9].
end
Use Bonferroni correction, BH [2], or BY [10] on $\{\widetilde{p}_j\}_{j=1}^{p}$ to call eGenes (the default is BY).

S2 Data simulation

In our simulation study, we approximately follow the data simulation in the second, more realistic simulation design of Zhou et al. [11], which approximately follows the data simulation in Wang et al. [12]. We simulate three data sets in total. Each data set is simulated according to Algorithm S5 with the following attributes:

- Sample size, $n = 838$.
- Number of genes, $p = 1000$.
- Number of covariates, $\widetilde{K} = 20$.
- Proportion of variance explained by genotype in eGenes, `PVEGenotype` $= 0.02$.
- Proportion of variance explained by covariates, `PVECovariates` $= 0.5$.

# of effect SNPs	Probability
0	0.35483532
1	0.34962617
2	0.18326554
3	0.07072812
4	0.02498728
5	0.01655758

Table S1: In our data simulation (Algorithm S5), the number of effect SNPs [11, 12] for each gene is sampled based on this probability table (the second column sums to one). This table is summarized from GTEx's independent cis-eQTL analysis [4] (see Figure S2 of Zhou et al. [11]). A gene is an eGene if and only if its number of effect SNPs is greater than zero.

Algorithm S5: Simulation of one data set

Randomly select p genes from GTEx's Brain - Cortex expression data [4], avoiding genes from the X chromosome [11, 12].

The goal is to simulate Y, the $n \times p$ gene expression matrix.

Simulate $\widetilde{X}$, the $n \times \widetilde{K}$ true covariate matrix, by drawing each entry independently from $N(0,1)$. In this work, all random sampling is independent unless otherwise specified.

for $j \leftarrow 1$ **to** p **do**

Obtain S_j, the $n \times q_j$ genotype matrix for gene j, by subsetting GTEx V8 genotype data [4]. Each column of S_j corresponds to a local common SNP for gene j. "Local" means the SNP is on the same chromosome as the gene and is located within one megabase (Mb) of the transcription start site (TSS) of the gene. "Common" means the minor allele frequency (MAF) of the SNP is at least 0.01 and the number of individuals with at least one copy of the minor allele (MA samples) is at least 10.

Sample $\widetilde{q}_j$, the number of effect SNPs [11, 12] for gene j, based on Table S1.

if $\widetilde{q}_j = 0$ **then**

Generate $Y[\,,j]$ based on

$$Y[\,,j] = \underset{n\times\widetilde{K}}{\widetilde{X}} \underset{\widetilde{K}\times 1}{\beta_{2j}} + \underset{n\times 1}{e_j}\,, \tag{S6}$$

where each entry of β_{2j} is drawn from $N(0,1)$, and each entry of e_j is drawn from $N(0,1)$ and scaled. The scaling is to ensure that `PVECovariates` is as desired. Specifically, we scale e_j so that

$$\frac{\mathrm{Var}(e_j)}{\mathrm{Var}\left(\widetilde{X}\beta_{2j}\right)} = \frac{1-\texttt{PVECovariates}}{\texttt{PVECovariates}}. \tag{S7}$$

else

Randomly select $\widetilde{q}_j$ columns of S_j and designate them as the effect SNPs of gene j.

Generate $Y[\,,j]$ based on

$$Y[\,,j] = \underset{n\times q_j}{S_j} \underset{q_j\times 1}{\beta_{1j}} + \underset{n\times\widetilde{K}}{\widetilde{X}} \underset{\widetilde{K}\times 1}{\beta_{2j}} + \underset{n\times 1}{e_j}\,, \tag{S8}$$

where entries of β_{1j} that don't correspond to the effect SNPs of gene j are set to 0, and entries of β_{1j} that correspond to the effect SNPs of gene j are each drawn from $N(0,1)$. Further, each entry of β_{2j} is drawn from $N(0,1)$ and scaled, and each entry of e_j is drawn from $N(0,1)$ and scaled. The scaling is to ensure that `PVEGenotype` and `PVECovariates` are as desired. Specifically, we scale β_{2j} so that

$$\frac{\mathrm{Var}\left(\widetilde{X}\beta_{2j}\right)}{\mathrm{Var}(S_j\beta_{1j})} = \frac{\texttt{PVECovariates}}{\texttt{PVEGenotype}} \tag{S9}$$

and separately scale e_j so that

$$\frac{\mathrm{Var}(e_j)}{\mathrm{Var}(S_j\beta_{1j})} = \frac{1-\texttt{PVEGenotype}-\texttt{PVECovariates}}{\texttt{PVEGenotype}}. \tag{S10}$$

end

end

A gene is an eGene if and only if its number of effect SNPs is greater than zero.

S3 Development of ClipperQTL

Here we describe the development of ClipperQTL.

Clipper [13] has four main technical parameters:

- Analysis: enrichment vs. differential analysis.
- Procedure: Barber-Candès (BC) vs. Gimenez-Zou (GZ) procedure.
- Contrast score: maximum vs. difference (i.e., minus) contrast score.
- h (only applicable under the GZ procedure, not the BC procedure).

In addition, in ClipperQTL, we can control B, the number of permutations (ClipperQTL terminology), i.e., the number replicates under the background condition (Clipper [13] terminology).

In ClipperQTL, we use enrichment analysis rather than differential analysis because in identifying eGenes, the alternative hypothesis is that the expectation of the maximum absolute correlation from the original expression data is *greater than* (rather than merely different from) the expectation of the maximum absolute correlation from permuted expression data.

In ClipperQTL, the number of replicates under the experimental condition is fixed at one because we only have one set of the original, unpermuted expression data. Therefore, if $B = 1$, then we only need to consider the BC procedure (in enrichment analysis, if the number of replicates under the experimental condition and the number of replicates under the background condition are both one, then the GZ procedure with either maximum or difference contrast score reduces to the BC procedure with difference contrast score); if $B > 1$, then we only need to consider the GZ procedure (in enrichment analysis, the BC procedure is only applicable when the number of replicates under the experimental condition and the number of replicates under the background condition are equal [13]). In other words, B determines the procedure we need to consider.

Therefore, we explore different combinations of B, contrast score, and h (only applicable under the GZ procedure). We find that for data sets with small sample sizes (< 450), no combination works well consistently, but for data sets with large sample sizes (> 450), two options work well: $B = 1$ and maximum contrast score; and $B \in [20, 100]$, maximum contrast score, and $h = 1$. These conclusions are drawn based on GTEx bulk data [4] and simulated data. Therefore, the Clipper variant of ClipperQTL is only recommended for data sets with large sample sizes (> 450), and the user is recommended to set $B = 1$ or B between 20 and 100. Regardless of the choice of B, enrichment analysis and maximum contrast score are used. If $B = 1$, the BC procedure is used; if $B \in [20, 100]$, the GZ procedure and $h = 1$ are used (these coincide with the default settings in Clipper [13] given B).

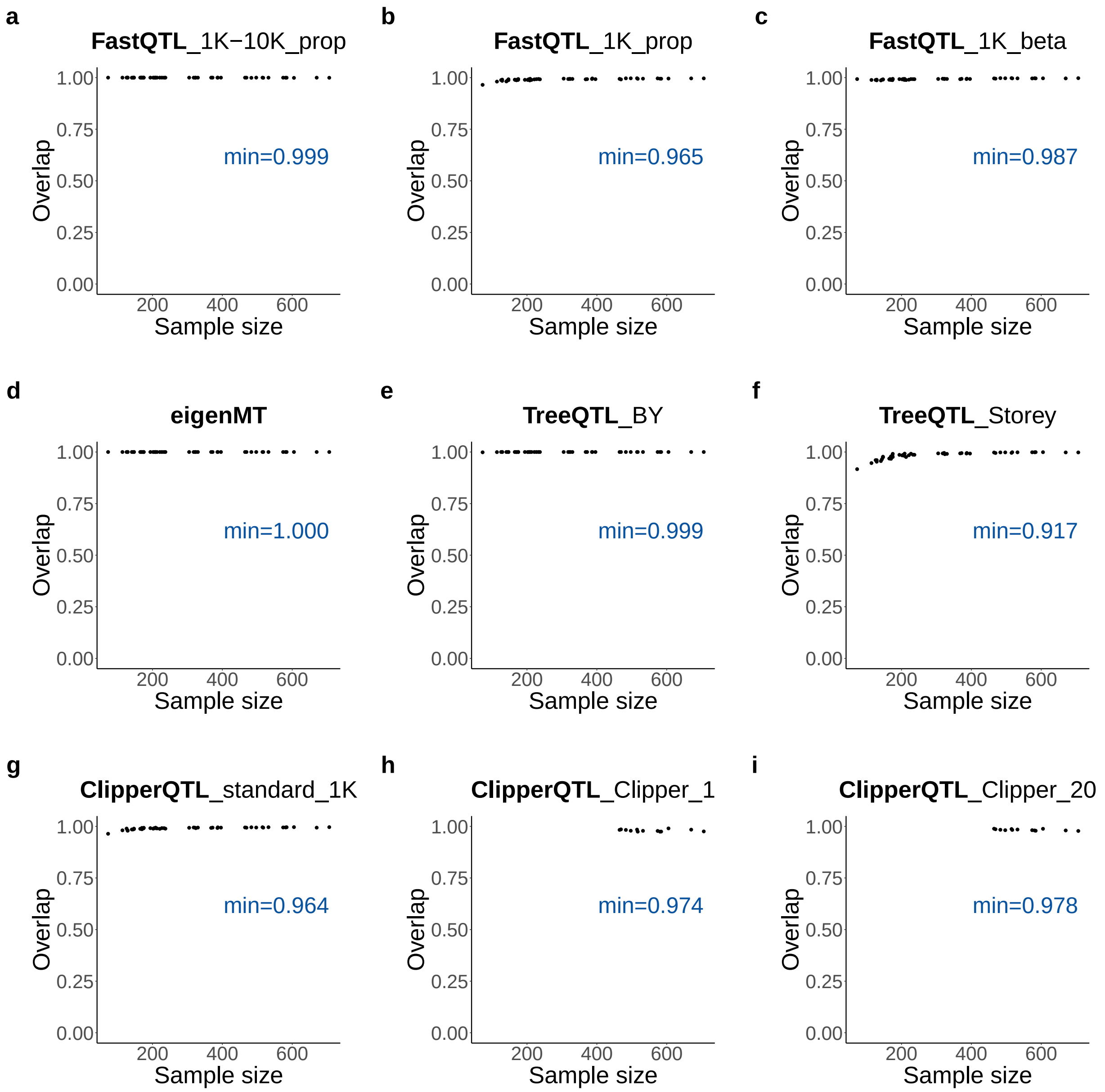

Figure S2: Overlap between eGenes identified by various methods and eGenes identified by FastQTL_1K-10K_beta—the default FastQTL method—in GTEx bulk data [4] (Table 1; see Section 5.3 for the analysis details). Each dot corresponds to a tissue. Given two sets, A and B, the overlap is defined as $|A \cap B| / \min(|A|, |B|)$, where $|\cdot|$ denotes the cardinality of a set. That is, the overlap between two sets is defined as the size of the intersection divided by the size of the smaller set. **b**, **c**, **g** The overlap is slightly lower when the sample size is smaller. This can be explained by the fact that power is generally lower when the sample size is smaller [4]. **h**, **i** Only tissues with sample sizes ≥ 465 are shown (Figure 1).

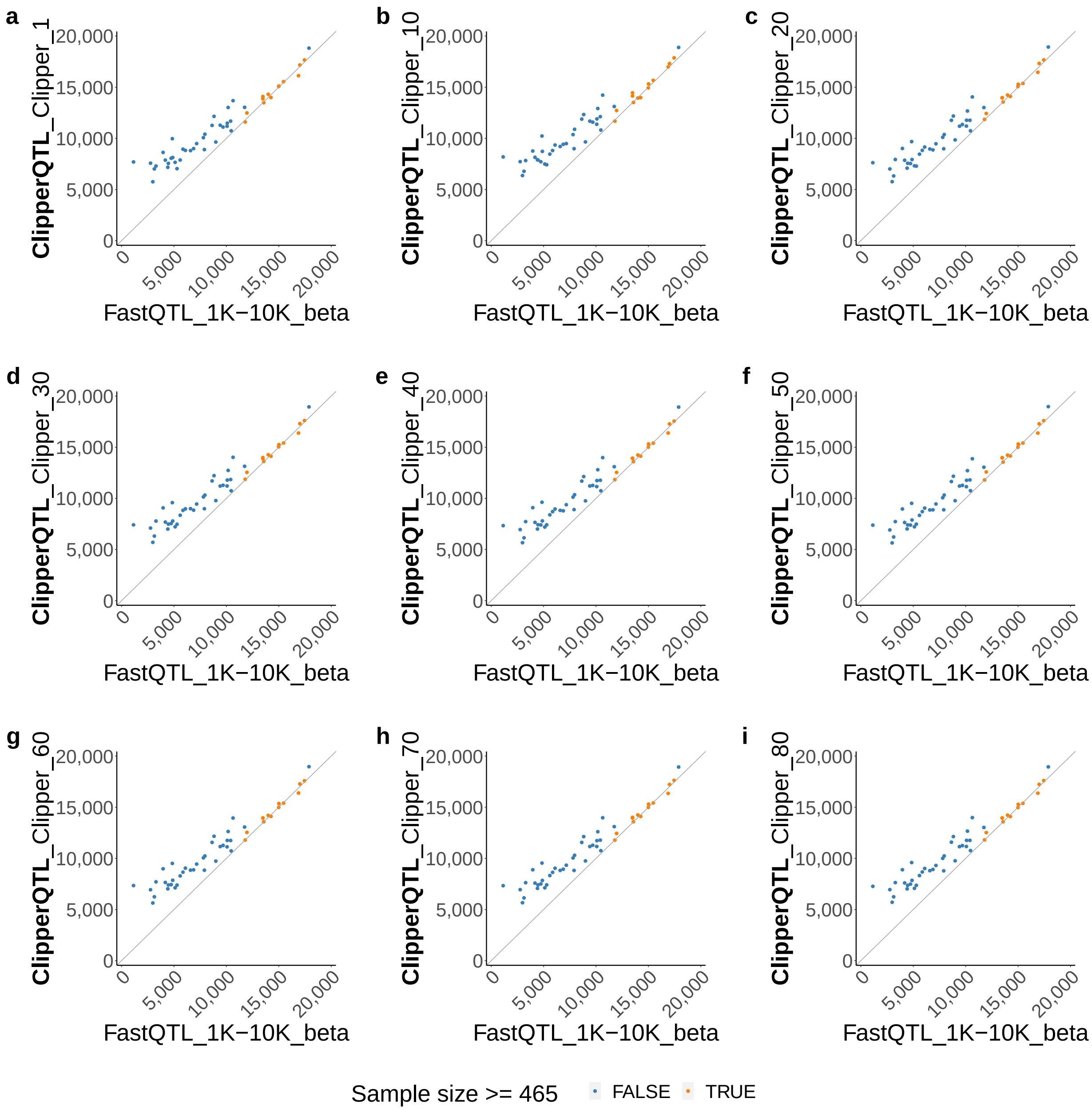

Figure S3: Number of eGenes comparison between ClipperQTL_Clipper with different B's and the default FastQTL method based on GTEx bulk data [4] (the analysis details are as described in Section 5.3). Each dot corresponds to a tissue. The x-axis and y-axis both represent numbers of eGenes identified by different methods. Diagonal lines through the origin are shown to help with visualization. Plots for ClipperQTL_Clipper_90 and ClipperQTL_Clipper_100 are omitted due to space limitation, but they look very similar to the plots shown here.

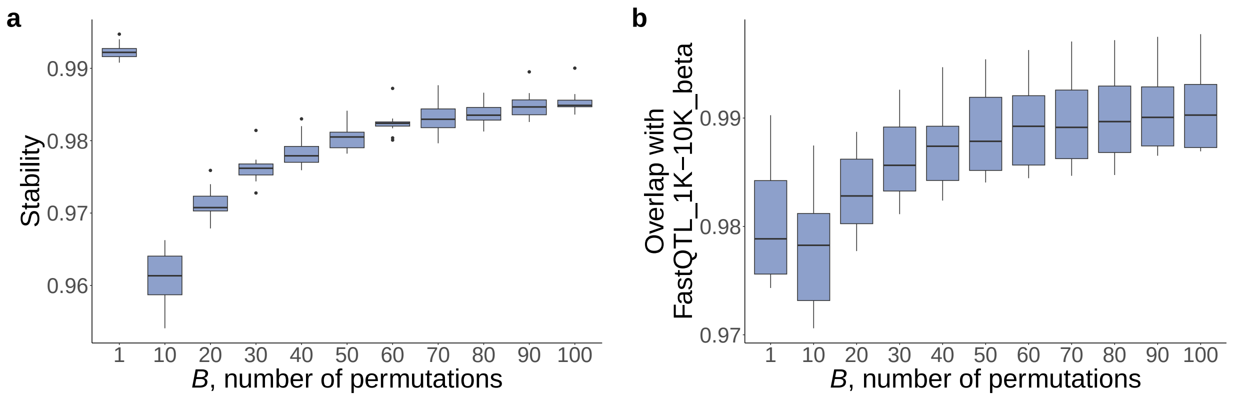

Figure S4: Comparison of ClipperQTL_Clipper with different B's. Each box plot contains 13 data points, corresponding to the 13 tissues in GTEx bulk data [4] with sample sizes ≥ 465 (the analysis details are as described in Section 5.3). See Figure S2 for our definition of overlap. Stability is a measure of how much the result of a method depends on the random seed; the higher the stability, the less the result varies with respect to the random seed. Specifically, to calculate the stability of a method (e.g., ClipperQTL_Clipper_1), we run the method 10 times with 10 different seeds. We divide the 10 runs into 5 pairs. For each pair, we calculate the overlap between the two sets of identified eGenes. The stability of the method is calculated as the average of the 5 overlaps.

References

[1] Andrey A. Shabalin. Matrix eQTL: Ultra fast eQTL analysis via large matrix operations. *Bioinformatics*, 28(10):1353–1358, 2012.

[2] Yoav Benjamini and Yosef Hochberg. Controlling the false discovery rate: A practical and powerful approach to multiple testing. *Journal of the Royal Statistical Society: Series B (Methodological)*, 57(1):289–300, 1995.

[3] Halit Ongen, Alfonso Buil, Andrew Anand Brown, Emmanouil T. Dermitzakis, and Olivier Delaneau. Fast and efficient QTL mapper for thousands of molecular phenotypes. *Bioinformatics*, 32(10):1479–1485, 2016.

[4] GTEx Consortium. The GTEx Consortium atlas of genetic regulatory effects across human tissues. *Science*, 369(6509):1318–1330, 2020.

[5] John D. Storey and Robert Tibshirani. Statistical significance for genomewide studies. *Proceedings of the National Academy of Sciences*, 100(16):9440–9445, 2003.

[6] Joe R. Davis, Laure Fresard, David A. Knowles, Mauro Pala, Carlos D. Bustamante, Alexis Battle, and Stephen B. Montgomery. An efficient multiple-testing adjustment for eQTL studies that accounts for linkage disequilibrium between variants. *The American Journal of Human Genetics*, 98(1):216–224, 2016.

[7] Olivier Ledoit and Michael Wolf. A well-conditioned estimator for large-dimensional covariance matrices. *Journal of Multivariate Analysis*, 88(2):365–411, 2004.

[8] C. B. Peterson, M. Bogomolov, Y. Benjamini, and C. Sabatti. TreeQTL: Hierarchical error control for eQTL findings. *Bioinformatics*, 32(16):2556–2558, 2016.

[9] R. J. Simes. An improved Bonferroni procedure for multiple tests of significance. *Biometrika*, 73(3):751–754, 1986.

[10] Yoav Benjamini and Daniel Yekutieli. The control of the false discovery rate in multiple testing under dependency. *The Annals of Statistics*, 29(4):1165–1188, 2001.

[11] Heather J. Zhou, Lei Li, Yumei Li, Wei Li, and Jingyi Jessica Li. PCA outperforms popular hidden variable inference methods for molecular QTL mapping. *Genome Biology*, 23(1):210, 2022.

[12] Gao Wang, Abhishek Sarkar, Peter Carbonetto, and Matthew Stephens. A simple new approach to variable selection in regression, with application to genetic fine mapping. *Journal of the Royal Statistical Society: Series B (Statistical Methodology)*, 82(5):1273–1300, 2020.

[13] Xinzhou Ge, Yiling Elaine Chen, Dongyuan Song, MeiLu McDermott, Kyla Woyshner, Antigoni Manousopoulou, Ning Wang, Wei Li, Leo D. Wang, and Jingyi Jessica Li. Clipper: P-value-free FDR control on high-throughput data from two conditions. *Genome Biology*, 22 (1):288, 2021.
